# Longitudinal Cerebral Perfusion Change in Transient Global Amnesia Related to Left Posterior Medial Network Disruption

**DOI:** 10.1371/journal.pone.0145658

**Published:** 2015-12-21

**Authors:** Jae-Won Jang, Young Ho Park, So Young Park, Min Jeong Wang, Jae-Sung Lim, Sung-Hun Kim, In KooK Chun, Youngsoon Yang, SangYun Kim

**Affiliations:** 1 Department of Neurology, Kangwon National University Hospital, Chuncheon, Republic of Korea; 2 Clinical Neuroscience Center, Seoul National University Bundang Hospital, Seongnam, Republic of Korea; 3 Department of Neurology, Seoul National University College of Medicine, Seoul, Korea; 4 Department of Neurology, Seoul National University Boramae Medical Center, Seoul, Korea; 5 Department of Nuclear Medicine, Kangwon National University Hospital, Chuncheon, Republic of Korea; 6 Department of Neurology, Seoul Veterans Hospital, Seoul, Korea; Hangzhou Normal University, CHINA

## Abstract

**Background:**

The pathophysiology of transient global amnesia (TGA) is not fully understood. Previous studies using single photon emission computed tomography (SPECT) have reported inconclusive results regarding cerebral perfusion. This study was conducted to identify the patterns of regional cerebral blood flow (rCBF) in TGA patients via longitudinal SPECT analysis. An association between the observed SPECT patterns and a pathophysiological mechanism was considered.

**Methods:**

Based on the TGA registry database of Seoul National University Bundang Hospital, 22 TGA patients were retrospectively identified. The subjects underwent initial Tc-99m-ethyl cysteinate dimer (ECD) SPECT within 4 days of an amnestic event and underwent follow-up scans approximately 6 months later. The difference in ECD uptake between the two scans was measured via voxel-based whole brain analysis, and the quantified ECD uptake was tested using a paired *t*-test.

**Results:**

The TGA patients had significantly decreased cerebral perfusion at the left precuneus (*P*<0.001, uncorrected) and at the left superior parietal and inferior temporal gyrus according to the voxel-based whole brain analysis (*P*<0.005, uncorrected). A difference in the quantified ECD uptake between the 2 scans was also found at the left precuneus among the 62 cortical volumes of interest (*P* = 0.018, Cohen’s *d* = -0.25).

**Conclusion:**

We identified left hemispheric lateralized hypoperfusion that may be related to posterior medial network disruption. These findings may be a contributing factor to the pathophysiology of TGA.

## Introduction

Transient global amnesia (TGA) refers to the sudden onset of anterograde and retrograde amnesia, which lasts up to 24 hours with no other cognitive impairment. TGA was first described more than 50 years ago [1], but the exact pathophysiological mechanism remains unknown. Several etiological factors, such as migraine, epilepsy, cerebral ischemia, venous flow abnormalities, have been proposed as possible explanations [2–7]. Among them, cerebral ischemia in terms of thromboembolic etiology or hemodynamics was investigated via several studies, but a definite association between TGA and arterial ischemia was not identified [8–14]. Instead of arterial ischemia, recent studies have reported venous congestion or venous reflux with subsequent ischemia combined with jugular valve insufficiency [4, 15–19]. However, such findings have also been thought of as ‘innocent bystanders’ due to the higher prevalence of jugular valve insufficiency without evidence of jugular venous reflux [16, 20, 21]. Therefore, the question remains as to how cerebral ischemia or hypoperfusion is related to the occurrence of TGA. Previous studies of cerebral hypoperfusion using single photon emission computed tomography (SPECT) have shown inconclusive results, as some describe medial temporal flow change [16, 22–25] and others report decreased or increased flow changes in various structures [24, 26–28]. This variability may be derived from the differences in the study designs, including imaging protocol and the latency of scanning. Most of studies have focused on the acute phase of attack and reported normalization with follow-up SPECT studies based on a few case observations, but longitudinal follow-up SPECT examinations using voxel-based whole brain analysis are rare. This study was conducted to identify the patterns of regional cerebral blood flow (rCBF) in TGA patients via longitudinal SPECT scans. We included TGA patients who underwent SPECT scans and analyzed the difference between the initial (within 4 days after symptom onset) and follow-up (at approximately 6 months after symptom onset) stages using voxel-based whole brain analysis and quantitative measurements of rCBF. To explore the natural evolution of the hemodynamic changes in TGA patients, we performed a retrospective analysis of the changes in cerebral perfusion measured via 99mTc-ethyl cysteinate dimer (99mTc-ECD) SPECT, which represented the rCBF. In addition, a possible association between the observed patterns of SPECT identified in this study and pathophysiological mechanisms is discussed.

## Materials and Methods

We prospectively collected consecutive patients who visited Seoul National University Bundang Hospital between October 2006 and December 2011 and who presented an acute episode of TGA. A diagnosis of TGA was made according to existing criteria [29, 30] as follows: (1) the presence of anterograde amnesia, which was witnessed by an observer, (2) no clouding of consciousness or a loss of personal identity, (3) cognitive impairment limited to amnesia, (4) no focal neurological or epileptic sign, (5) no recent history of head trauma or seizures, (6) resolution of symptoms within 24 h, and (7) mild headache, nausea or dizziness, which may be present during the acute phase. Through the retrospective analysis using this prospective registry database, we collected TGA patients who had underwent 2 SPECT examinations with an interval of approximately 6 months, and a total of 22 patients were identified. Although TGA symptoms last less than 24 hrs, the cerebral hypoperfusion observed in SPECT could persist up to several months, and therefore, we performed follow-up scans at approximately 6 months, which was regarded as a sufficient time interval for patients to recover from relatively sustained hypoperfusion [26, 27, 31–33]. All of the subjects were examined for demographics and clinical findings from the medical registry database. MRI and SPECT images were read by board-certified neuroradiologists and nuclear medicine physicians, respectively. This study protocol was approved by the Seoul National University Bundang Hospital institutional review board, with an informed consent waiver due to the minimal risk to participants and the study’s retrospective nature. The record/information of patient was anonymized and de-identified prior to analysis.

### Imaging parameters

SPECT images were obtained using a triple-head gamma camera (Trionix Triad; Trionix Research Laboratory, Inc., Twinsburg, OH) equipped with a low-energy, fan-beam collimator. Patients were instructed to close their eyes in a dimly lit room with minimal background noise. Scanning was initiated 10 minutes after an intravenous injection of 15 mCi of Tc-99m-ethylcysteine dimer (ECD). The data were acquired in a 128x128 matrix with a voxel size of 1.7861.7861.78 mm and then reconstructed via filtered back-projection using a Butterworth filter (cutoff frequency 0.6 cycle per cm, order 8) to reduce statistical noise. The correction for tissue attenuation was also performed.

MRI was performed within a few hours after arriving at the hospital using either a 1.5-Tesla unit (Intera; Philips Medical Systems, Best, the Netherlands) or a 3-Tesla unit (intera Achieva; Philips, Best, the Netherlands) with a sensitivity encoding (SENSE) head coil. The diffusion-weighted imaging (DWI) that was included in the MRI protocol [34] was performed again at the 3^rd^ day from onset according to the same protocol. Single-shot spin-echo echo-planar imaging was used for DWI using following parameters: a matrix 128x128 interpolated to 256x256; field of view, 220 mm; repetition time, 9400 ms for the 1.5 Tesla and 5000 ms for the 3 Tesla; echo time, 66 ms for the 1.5 Tesla and 59 ms for the 3 Tesla; SENSE factor, 2; number of acquisitions, 4; b value, 2000 s/mm^2^; and section thickness, 3 mm.

### SPM and image data analysis of cerebral perfusion

SPECT images were analyzed using SPM8 (Institute of Neurology, University College London, London, UK) [20], which was implemented using Matlab 2009a (The MathWorks Inc., Natick, MA). The mean voxel intensity across all slices of the imaging volume was calculated. Each voxel was then thresholded at 80% of the mean intensity to eliminate background noise and partial volume effects at the edge of the brain [35, 36]. Each SPECT scan was then spatially normalized to the SPECT template provided by SPM8 using an affine geometric transformation. These images were smoothed using an isotropic Gaussian kernel of 12 mm full width at half maximum. Then, a proportional scaling was applied to remove the effect of differences in global activity. After normalization and smoothing, a paired comparison of the SPECT images from each patient at the baseline and follow-up studies were performed on a voxel-by-voxel based paired t statistic. We investigated areas with hypoperfusion at a threshold of *P* <0.005 (uncorrected) and an extent threshold of 100 voxels. To visualize the result, the significant voxels were projected onto the ch2better template included in MRIcron (http://www.mccauslandcenter.sc.edu/mricro/mricron/). To evaluate the quantitative analysis, we measured the ECD uptake in regional volume of interest (VOI) according to the Automated Anatomical Labelling (AAL) merged template using Matlab. Among the 71 regions in the AAL-merged atlas, 62 regions were selected after excluding 9 regions of the posterior fossa. To quantify the regional ECD uptake, the mean counts in each selected region were normalized with respect to the mean count in the cerebellum, which is known to be a relatively stable area. Because ECD SPECT does not estimate absolute quantitative blood flow values but rather provides relative regional flow differences among various regions, we selected the cerebellum as the reference for normalization [37]. Then, the mean differences were obtained by subtracting the relative ECD uptake counts between two scans. We calculated the effect size using Cohen’s *d* values [38]. In addition, a paired Student’s *t* test was used to evaluate the differences in the relative mean count of the regional CBF between baseline and follow-up using SPSS version 18.0 (SPSS Inc., Chicago, IL, USA). A Wilcoxon signed rank test was used to compare the variables that did not meet normality. Statistical significance was set at p<0.05.

## Results

**Fig 1:**
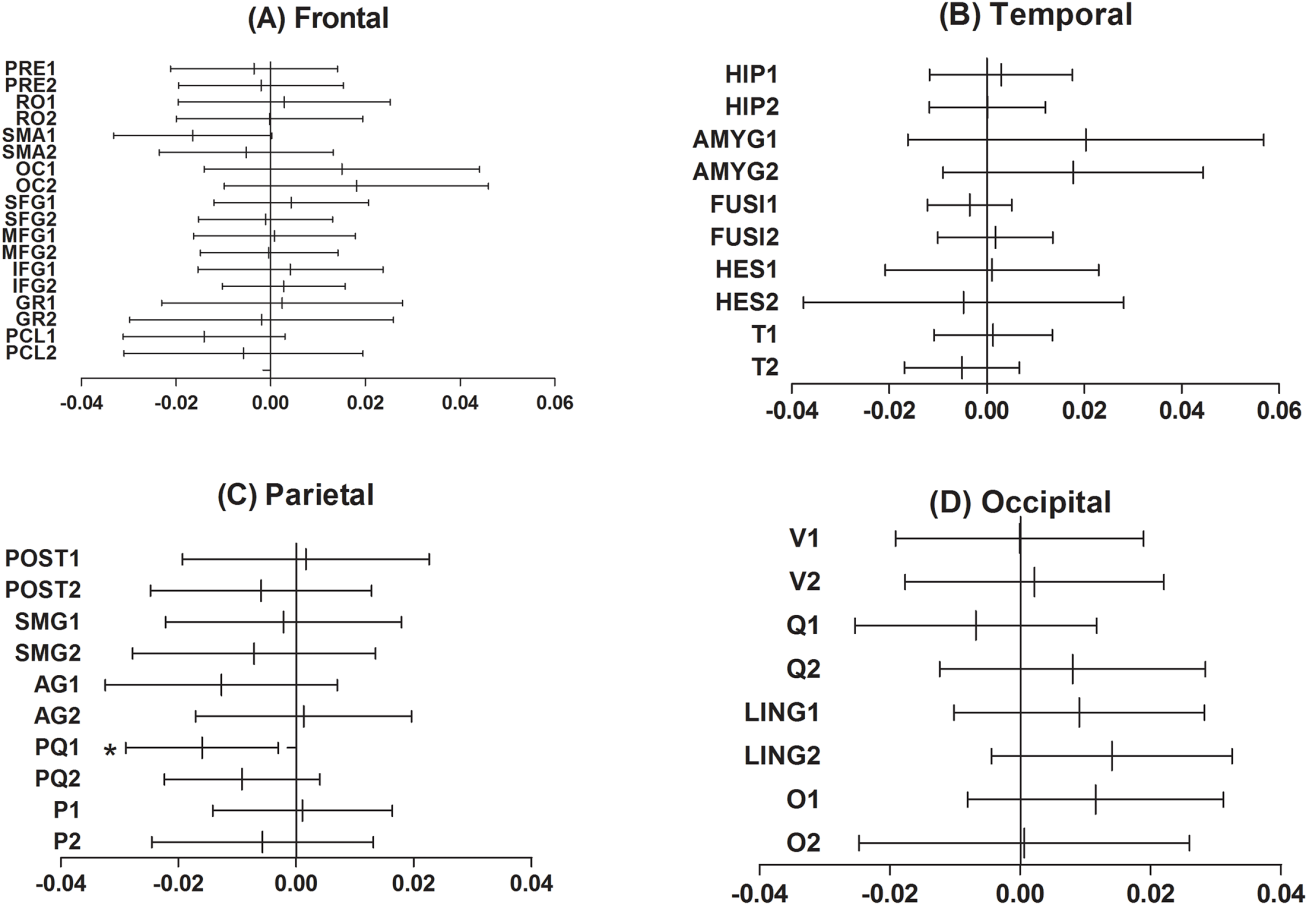
The mean difference in the regional cerebral perfusion between the initial and follow-up stages of TGA. Only the left precuneus exhibited a significant difference between the 2 scans among the 62 volumes of interest (VOIs) (*P* = 0.018, Cohen’s *d* = -0.25). The VOI of the central structures, the insula and the cingulate gyrus, which showed no significant differences between the 2 scans, are not presented here. The mean difference was obtained by subtracting the ECD uptake of the follow-up scan from the initial scans and demonstrated by the VOIs according to the cortical lobes. The vertical line in the center of the each horizontal line represents the mean difference, and the whiskers indicate the 95% confidence interval. Abbreviation: 1,left; 2,right; PRE, precentral gyrus; RO, roalandic operculum; SMA, supplementary motor area; OC, olfactory cortex; SFG, superior frontal gyrus; MFG, middle frontal gyrus; GR, gyrus rectus; PCL, paracentral lobule; HIP, hippocampus; AMYG, amygdala; FUSI, fusiform gyrus; HES, Heschl gyrus; T, temporal gyrus; POST, postcentral gyrus; SMG, supramarginal gyrus; AG, angular gyrus; PQ, precuneus; P, superior and inferior parietal lobule; V, calcarine fissure and surrounding cortex; Q, cuneus; LING, lingual gyrus; O, lateral remainder of occipital lobe.

**Fig 2:**
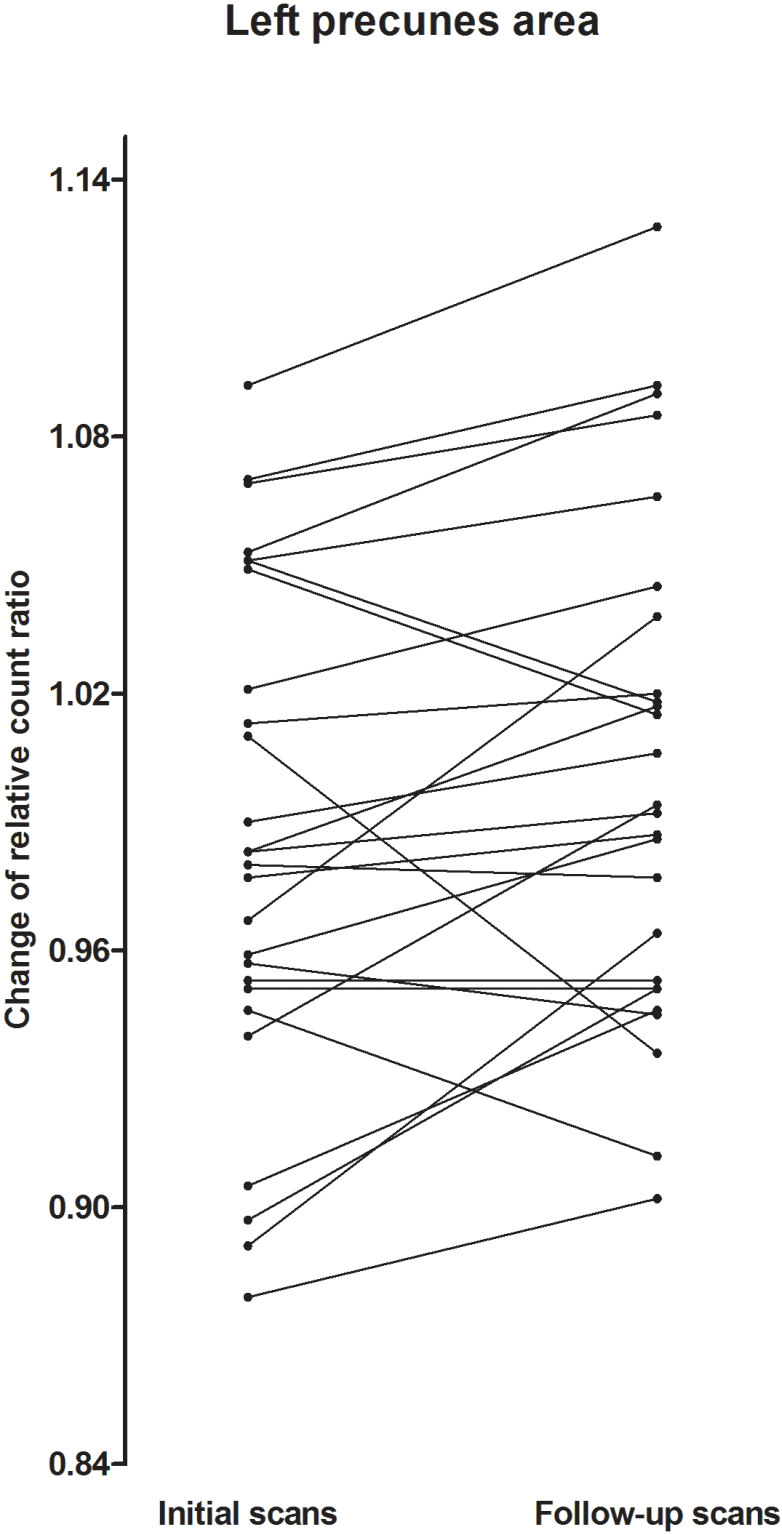
Changes in ECD uptake counts across the 6-month-interval of the TGA patients at the left precuneus. The ECD count of each patient was plotted to demonstrate the change between the two scans using a paired Student’s *t* test.

**Fig 3:**
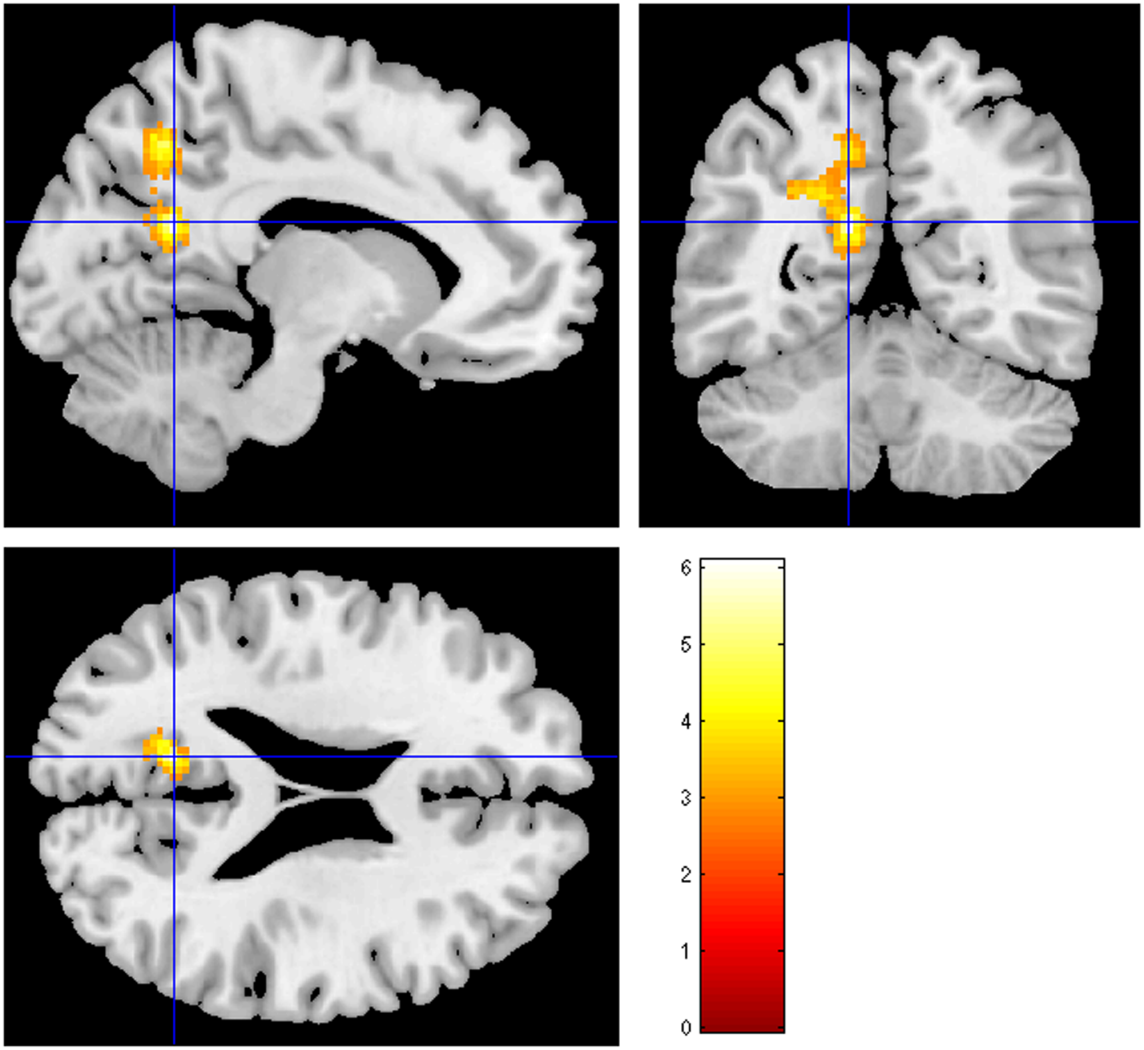
Statistical parametric mapping projections of areas with significantly reduced cerebral perfusion when comparing the follow-up SPECT scans with the initial scans (Uncorrected *p*<0.005). The warm color represents the relative hypoperfusion of the initial scans compared to the follow-up scans.

The clinical characteristics of the study participants are summarized in Table 1. Based on visual readings, the initial SPECT hypoperfusion revealed no laterality and was significant in medial temporal areas in most of the patients. The number of detected bilateral TGA specific DWI lesions was higher than the number of unilateral lesions, which also revealed no definite laterality. The mean differences in the relative CBF count between the initial and follow-up scans in TGA patients were demonstrated by the cortical regions (Fig 1). Among the 62 cortical VOIs, only the left precuneus exhibited significant a difference between the 2 scans (*P* = 0.018, Cohen’s *d* = -0.25). Compared to the follow-up scan, the ECD uptake was significantly decreased in the left precuneus (Fig 2). This finding was also observed in the voxel-based whole brain analysis (Fig 3), which revealed decreased cerebral perfusion in the left precuneus (uncorrected *p*<0.001) and in the left inferior temporal and superior parietal areas (uncorrected *p*<0.005). The areas with most pronounced decrease in regional cerebral perfusion between the initial scans and follow-up scans are listed in Table 2. No cerebral regions exhibited a significant increase in perfusion between the initial and follow-up scans.

**Table 1:** Baseline Characteristics of the study population (n = 22).

Age in years, mean (SD)	60.55 (6.36)
Males, n (%)	6 (27.3)
Duration of TGA in hours, mean (SD)	5.81 (3.92)
Hypertension, n (%)	8 (36.4)
Diabetes, n (%)	3 (13.7)
Hyperlipidemia, n (%)	11 (50)
Precipitating factor, n (%)	
Physical stress	9 (40.9)
Emotional stress	8 (36.4)
Water contact	2 (9.1)
Valsalva	2 (9.1)
Associated symptom, n (%)	
Headache	5 (22.7)
Nausea	3 (13.6)
Days from symptom onset to SPECT study	
Initial SPECT, mean (SD)	1.49 (1.37)
Follow up SPECT, mean (SD)	166.48 (89.08)
Relative laterality of initial SPECT abnormality, n (%)	
Left	9 (40.9)
Right	9 (40.9)
Bilateral	4 (18.2)
Location of initial SPECT abnormality, n (%)	
Medial temporal	21 (95.5)
Neocortex	4 (18.2)
Sub-cortex	1 (4.5)
Laterality of DWI lesion, n (%)	
Left	3 (13.6)
Right	4 (18.2)
Bilateral	9 (40.9)
negative	7 (31.8)

Abbreviations: SPECT, Single-photon emission computed tomography; DWI, diffusion-weighted imaging; SD, standard deviation.

**Table 2:** Location and peaks of significant decreases in regional cerebral perfusion from the baseline to follow-up scans (Uncorrected p<0.005).

Structure	Talairach coordinates			p value (uncorrected)	Z-score
	x	y	z		
Left precuneus	-12	-56	22	<0.001	4.57
Left superior parietal	-18	-60	52	<0.001	3.70
Left inferior temporal	-38	2	-36	0.002	2.86

## Discussion

In this study, we retrospectively analyzed longitudinal changes in the cerebral perfusion of TGA patients based on two SPECT scans performed at six-month intervals. Cerebral perfusion was reduced the greatest in the left precuneus at baseline compared to the follow-up scans for both the voxel-based whole brain analysis and the regional quantitative ECD uptake. To the best of our knowledge, this is the first and largest study that compared SPECT changes between two stages using both voxel-based whole brain analysis and quantitative ECD uptake counts analyzed using paired t tests.

In regard to reversible hypoperfusion in the left precuneus, we postulated that this finding may also reflect transient abnormal cerebral perfusion during the acute phase, and it recovered to a normal state afterwards. One possible explanations is abnormal cerebral venous drainage, which is the leading hypothesis of the underlying pathophysiologic mechanisms of TGA [39–42]. Although there remain many unsolved questions, patients with TGA have exhibited more prevalent venous abnormalities in several studies of retrograde venous flow [15, 21, 39, 40]. One possibility is that precipitating factors, such as Valsalva-like episodes, could induce intracranial venous hypertension and may trigger reactive arterial vasoconstriction to compensate for the increased cerebral blood volume. In addition, the subsequent relative hypoperfusion may be responsible for the amnestic event in susceptible individuals [41] because of the selective vulnerability of CA1 (cornu ammonis) region to ischemic insult[43]. In addition to these previous studies, our longitudinal comparison of cerebral perfusion yields additional information regarding the hypoperfusion of precuneus.

When we assume that the precuneus region belongs to the posterior medial network [44] for the conformation of episodic memory, these findings are compatible with our previous study based on an electroencephalographic (EEG) spectral analysis, which also implicated the posterior medial network disruption during acute phase of TGA [45]. It was recently reported that if the posterior medial network connecting the posterior cortical areas and the hippocampus is disrupted, episodic memory is impaired [46]. Therefore, we speculated that hypoperfusion of the precuneus as a representative of altered posterior medial network during initial stage of TGA may be related to the amnesia of TGA patients. This idea of ‘two cortical systems’ for memory was recently proposed as the anterior temporal system made up of the perirhinal cortex and posterior medial system, which includes the parahippocampal cortex, the retrosplenial cortex and the default network (composed of the posterior cingulate, the precuneus, the lateral parietal cortex and the medial prefrontal cortex) [47, 48]. This framework of two interacting systems extends the connection of the hippocampus beyond the medial temporal lobes. In addition, it explains the differences in the functional and anatomical connectivity of the medial temporal lobe with a distinct contribution to memory. Evidence has consistently suggested that the posterior medial network is involved in episodic memory [49–51]. Therefore, hypoperfusion at the precuneus, which is the primary component of the posterior medial system, may have implications in the interactions regarding hippocampal formation. Although the disruptions in the structural connectivity of the limbic system were not identified[52], the approach based on the functional network has also been investigated via resting-state functional MRI, which revealed a significant reduction in the functional connection of the episodic memory network during the ictal phase of TGA [53].

In addition, the voxel-based whole brain analysis also revealed cerebral hypoperfusion in the left inferior temporal and superior parietal cortex and in the left precuneus. This finding of left lateralization is in line with other studies that reported some left dominance of functional abnormalities confirmed via EEG, perfusion MRI or SPECT abnormalities [27, 28, 54, 55]. Considering that visual reading of SPECT images and DWI lesions are not related to left dominance (Table 1), there may be some discrepancies from the findings of the voxel-based whole brain analysis or the quantitative ECD uptake. One possible explanation is that the effect of left cerebral hypoperfusion contributing to amnestic events in TGA patients could be greater or persist longer than that of the right side, although bilateral cerebral hypoperfusion may occur at the initial stage of TGA. We speculated that this finding regarding left laterality could be closely linked to a previous study reported by our team, which revealed significant left predominance of EEG abnormalities [54].

Previous reported findings using SPECT imaging of TGA patients were inconsistent, and most performed region-of-interest analyses based on visual readings of a few cases (Table 3). However, most of the studies revealed bilateral or left cerebral hypoperfusion, although the right side was related to hyperperfusion in some studies [26, 36]. A study using SPM was previously conducted, although it differs from this study [36]. The design of the previous study was based on a case-control comparison of seven TGA patients and age-matched controls, whereas this study focused on observations of longitudinal changes in ECD uptake in TGA patients at 6-month intervals. This intra-individual change in ECD uptake adds another aspect of hemodynamic changes in TGA patients, as described above. In addition, the results of the SPM analysis were compared with the quantitative analysis of ECD uptake, revealing results that were consistent with hypoperfusion of the left precuneus. Therefore, the findings in our study may be applicable, as our data were confirmed by two separate methods and utilized a larger patient group. Although group analysis showed increased ECD uptake count of left precuneus at resolved stage, decreased uptake was also found in some patients that was different from other patients (Fig 2). These findings might be attributable to variability of patient characteristics such as degree of ischemia during initial scan, timing of SPECT acquisition and underlying degenerative pathology that could cause hypoperfusion.

**Table 3:** Previous SPECT studies investigated cerebral perfusion of TGA patient.

Author (year)	Patient (No)	SPECT protocol	Study design	Results
Stillhard (1990)[57]	1	99m-Tc-HMPAO	Case report	Reversible bitemporal hypoperfusion
		Visual reading	Longitudinal f/u	
Gondenberg (1991)[31]	1	99m-Tc-HMPAO	Case report	Reversible bilateral thalamic (left>right) hypoperfusion
		Quantitative analysis	Longitudinal f/u	Persistent left frontal hypoperfusion
Evans (1993)[22]	1	99m-Tc-HMPAO	Case report	Reversible bilateral postero-medial hypoperfusion
		Visual reading	Longitudinal f/u	
Lin (1993)[58]	1	99m-Tc-HMPAO	Case report	Reversible multiple perfusion defects in both occipital lobes, the medial left temporal lobe, and the left thalamus
		Visual reading	Longitudinal f/u	
Jung (1996)[26]	1	99m-Tc-ECD	Case report	Reversible hyperperfusion in the posterior caudal region of the right medial temporal lobe
		Visual reading	Longitudinal f/u	
		Quantitative analysis (ROI)		
Jovin (2000)[24]	1	99m-Tc-bicisate	Case report	Reversible bilateral medial temporal hypoperfusion
		Visual reading	Longitudinal f/u	
Laloux (1992) [27]	5	99m-Tc-HMPAO	Case series	
		Visual reading	Cross-sectional	Left temporal, parietotemporal hypoperfusion
			Longitudinal f/u	Persistent left temporal hypoperfusion
Matsuda (1993)[28]	1	99m-Tc-HMPAO	Case report	Reversible left medial temporal hyperperfusion
		Visual reading	Longitudinal f/u	
Sakashita (1997)[59]	6	99m-Tc-HMPAO	Case series	Reversible occipital and cerebellar hyperperfusion
		Visual reading	Longitudinal f/u	Reversible thalamic, cerebellar or putamenal hypoperfusion
Takeuch (1998)[60]	1	99m-Tc-ECD with acetazolamide (ACZ)	Case report	Reversible left medial temporal and thalamic poor vasodilatory reactivity to ACZ and hypoperfusion
		Visual reading	Longitudinal f/u	
Nardone (2004)[61]	13	99m-Tc-HMPAO	Case series	Reversible or persistent thalamic, striatal or temporal hypoperfusion
		Visual reading	Longitudinal f/u	
Chung (2009)[36]	7	99m-Tc-ECD	Case-control study	Reversible bilateral inferior, middle frontal hypoperfusion
		Voxel-based whole brain analysis	Longitudinal f/u	Reversible superior temporal, precentral and postcentral hypoperfusion (left>right)
				Reversible middle, superior temporal, inferior frontal, cerebellar and thalamic hyperperfusion (right>left)
Yamane (2008)[33]	1	^123^I-IMP	Case report	Reversible diffuse cerebral hypoperfusion
		Visual reading	Longitudinal f/u	
Current study	22	99m-Tc-ECD	Longitudinal f/u	Hypoperfusion in the left precuneus, inferior temporal and superior parietal area.
		Voxel-based whole brain analysis		
		Quantitative analysis (VOI)		

SPECT, Single-photon emission computed tomography; 99m-Tc-HMPAO, hexamethylpropyleneamineoxim; 99m-Tc-ECD, ethyl cysteinate dimer; ROI, region of interest; VOI, volume of interest;

^123^I-IMP, Iodine-123-labeled N-isopropyl-4-iodoamphetamine.

There are several limitations in this study. First, we could not compare the ECD uptake between TGA patients and normal controls because normal SPECT images were not available. Second, a detailed neuropsychological test was unavailable during the two stages of TGA, and therefore, we could not confirm the relationship between amnestic syndrome and the findings of our SPECT study. Third, initial SPECT images were not obtained during the ictal stage of TGA, and an interval of 6 months for the follow-up scan may not be sufficient time for patients to recover fully from the initial hypoperfusion state; therefore, it is possible that real perfusion changes could be more extensive than our findings if we expand the interval of the 2 scans. Fourth, the significance level was not corrected for multiple comparisons in the SPM analysis due to the small sample size. Thus, we utilized a cluster filter of 100 voxels to reduce the false positive identification rate [56].

In conclusion, we identified left hemispheric lateralized hypoperfusion at the initial stage of TGA using longitudinal SPECT scans, which may be related to posterior medial network disruption with left dominancy, in connection with recent studies [45, 54].

## Supporting Information

S1 Table Raw Data Set for TGA Study. (XLSX)

## References

[1] Fisher CM, Adams RD. TRANSIENT GLOBAL AMNESIA. Acta neurologica Scandinavica Supplementum. 1964;40:SUPPL 9:1–83. Epub 1964/01/01. . 14198929

[2] Sander K, Sander D. New insights into transient global amnesia: recent imaging and clinical findings. 2005;4(7):437–44. 10.1016/S1474-4422(05)70121-6 . 15963447

[3] Bartsch T, Alfke K, Deuschl G, Jansen O. Evolution of hippocampal CA-1 diffusion lesions in transient global amnesia. 2007;62(5):475–80. 10.1002/ana.21189 . 17702037

[4] Lewis SL. Aetiology of transient global amnesia. Lancet. 1998;352(9125):397–9. Epub 1998/08/26. 10.1016/s0140-6736(98)01442-1 . 9717945

[5] Yang Y, Kim S, Kim JH. Ischemic evidence of transient global amnesia: location of the lesion in the hippocampus. Journal of clinical neurology (Seoul, Korea). 2008;4(2):59–66. Epub 2009/06/11. 10.3988/jcn.2008.4.2.59 19513305 PMC2686867

[6] Donnet A. Transient Global Amnesia Triggered by Migraine in a French Tertiary-Care Center: An 11-Year Retrospective Analysis. Headache. 2015;55(6):853–9. Epub 2015/04/17. 10.1111/head.12545 . 25877480

[7] Lin KH, Chen YT, Fuh JL, Li SY, Chen TJ, Tang CH, et al Migraine is associated with a higher risk of transient global amnesia: a nationwide cohort study. Eur J Neurol. 2014;21(5):718–24. Epub 2014/02/14. 10.1111/ene.12346 . 24520813

[8] Hodges JR, Warlow CP. The aetiology of transient global amnesia. A case-control study of 114 cases with prospective follow-up. 1990;113 (Pt 3):639–57. . 2194627 10.1093/brain/113.3.639

[9] Pantoni L, Bertini E, Lamassa M, Pracucci G, Inzitari D. Clinical features, risk factors, and prognosis in transient global amnesia: a follow-up study. 2005;12(5):350–6. 10.1111/j.1468-1331.2004.00982.x . 15804264

[10] Winbeck K, Etgen T, von Einsiedel HG, Rottinger M, Sander D. DWI in transient global amnesia and TIA: proposal for an ischaemic origin of TGA. 2005;76(3):438–41. 10.1136/jnnp.2004.042432 15716545 PMC1739538

[11] Melo TP, Ferro JM, Ferro H. Transient global amnesia. A case control study. Brain. 1992;115 Pt 1:261–70. . 1559158 10.1093/brain/115.1.261

[12] Zorzon M, Antonutti L, Mase G, Biasutti E, Vitrani B, Cazzato G. Transient global amnesia and transient ischemic attack. Natural history, vascular risk factors, and associated conditions. Stroke. 1995;26(9):1536–42. . 7660394 10.1161/01.str.26.9.1536

[13] Enzinger C, Thimary F, Kapeller P, Ropele S, Schmidt R, Ebner F, et al Transient global amnesia: diffusion-weighted imaging lesions and cerebrovascular disease. 2008;39(8):2219–25. 10.1161/STROKEAHA.107.508655 . 18583561

[14] Baracchini C, Farina F, Ballotta E, Meneghetti G, Manara R. No signs of intracranial arterial vasoconstriction in transient global amnesia. Journal of neuroimaging: official journal of the American Society of Neuroimaging. 2015;25(1):92–6. Epub 2014/02/28. 10.1111/jon.12090 . 24571186

[15] Agosti C, Borroni B, Akkawi NM, Padovani A. Cerebrovascular risk factors and triggers in transient global amnesia patients with and without jugular valve incompetence: results from a sample of 243 patients. 2010;63(5):291–4. 10.1159/000292502 . 20413973

[16] Baracchini C, Tonello S, Farina F, Viaro F, Atzori M, Ballotta E, et al Jugular veins in transient global amnesia: innocent bystanders. Stroke. 2012;43(9):2289–92. Epub 2012/07/20. 10.1161/strokeaha.112.654087 . 22811457

[17] Akkawi NM, Agosti C, Rozzini L, Anzola GP, Padovani A. Transient global amnesia and disturbance of venous flow patterns. Lancet. 2001;357(9260):957 10.1016/S0140-6736(05)71655-X . 11289371

[18] Yang Y, Kim JS, Kim S, Kim YK, Kwak YT, Han IW. Cerebellar Hypoperfusion during Transient Global Amnesia: An MRI and Oculographic Study. Journal of clinical neurology (Seoul, Korea). 2009;5(2):74–80. Epub 2009/07/10. 10.3988/jcn.2009.5.2.74 19587813 PMC2706414

[19] Kim J, Kwon Y, Yang Y, Jang IM, Chang Y, Park YH, et al Clinical Experience of Modified Diffusion-Weighted Imaging Protocol for Lesion Detection in Transient Global Amnesia: An 8-Year Large-Scale Clinical Study. Journal of neuroimaging: official journal of the American Society of Neuroimaging. 2013 Epub 2013/04/05. 10.1111/jon.12021 . 23551898

[20] Kang Y, Kim E, Kim JH, Choi BS, Jung C, Bae YJ, et al Time of flight MR angiography assessment casts doubt on the association between transient global amnesia and intracranial jugular venous reflux. European radiology. 2014 Epub 2014/10/04. 10.1007/s00330-014-3448-7 . 25278248

[21] Lochner P, Nedelmann M, Kaps M, Stolz E. Jugular valve incompetence in transient global amnesia. A problem revisited. Journal of neuroimaging: official journal of the American Society of Neuroimaging. 2014;24(5):479–83. Epub 2013/09/17. 10.1111/jon.12042 . 24033644

[22] Evans J, Wilson B, Wraight EP, Hodges JR. Neuropsychological and SPECT scan findings during and after transient global amnesia: evidence for the differential impairment of remote episodic memory. Journal of neurology, neurosurgery, and psychiatry. 1993;56(11):1227–30. Epub 1993/11/01. ; PubMed Central PMCID: PMCPmc489828. 8229038 10.1136/jnnp.56.11.1227 PMC489828

[23] Schmidtke K, Reinhardt M, Krause T. Cerebral perfusion during transient global amnesia: findings with HMPAO SPECT. Journal of nuclear medicine: official publication, Society of Nuclear Medicine. 1998;39(1):155–9. Epub 1998/01/27. . 9443755

[24] Jovin TG, Vitti RA, McCluskey LF. Evolution of temporal lobe hypoperfusion in transient global amnesia: a serial single photon emission computed tomography study. Journal of neuroimaging: official journal of the American Society of Neuroimaging. 2000;10(4):238–41. Epub 2001/01/09. . 11147408 10.1111/jon2000104238

[25] Warren JD, Chatterton B, Thompson PD. A SPECT study of the anatomy of transient global amnesia. Journal of clinical neuroscience: official journal of the Neurosurgical Society of Australasia. 2000;7(1):57–9. Epub 2000/06/10. 10.1054/jocn.1998.0129 . 10847653

[26] Jung HH, Baumgartner RW, Burgunder JM, Wielepp JP, Lourens S, Wielepp JP. Reversible hyperperfusion of the right medial temporal lobe in transient global amnesia. Journal of neurology, neurosurgery, and psychiatry. 1996;61(6):654–5. Epub 1996/12/01. ; PubMed Central PMCID: PMCPmc486671. 8971124 10.1136/jnnp.61.6.654-a PMC486671

[27] Laloux P, Brichant C, Cauwe F, Decoster P. Technetium-99m HM-PAO single photon emission computed tomography imaging in transient global amnesia. Archives of neurology. 1992;49(5):543–6. Epub 1992/05/01. . 1580818 10.1001/archneur.1992.00530290131022

[28] Matsuda H, Higashi S, Tsuji S, Sumiya H, Miyauchi T, Hisada K, et al High resolution Tc-99m HMPAO SPECT in a patient with transient global amnesia. Clinical nuclear medicine. 1993;18(1):46–9. Epub 1993/01/01. . 8422720 10.1097/00003072-199301000-00011

[29] Caplan L. Transient global amnesia. In: Vinken PJ, Bruyn GW, Klawans HL, eds. Amsterdam, Netherlands: Elsevier Science Publishing Co; 1985 205–18 p.

[30] Hodges JR, Warlow CP. Syndromes of transient amnesia: towards a classification. A study of 153 cases. 1990;53(10):834–43. 2266362 10.1136/jnnp.53.10.834 PMC488242

[31] Goldenberg G, Podreka I, Pfaffelmeyer N, Wessely P, Deecke L. Thalamic ischemia in transient global amnesia: a SPECT study. Neurology. 1991;41(11):1748–52. Epub 1991/11/01. . 1944904 10.1212/wnl.41.11.1748

[32] Caffarra P, Concari L, Gardini S, Spaggiari S, Dieci F, Copelli S, et al Recovery from transient global amnesia following restoration of hippocampal and fronto—cingulate perfusion. Behavioural neurology. 2010;22(3–4):131–9. Epub 2009/01/01. 10.3233/ben-2009-0253 . 20595745 PMC5434422

[33] Yamane Y, Ishii K, Shimizu K, Sofue K, Yoshikawa T, Miyamoto N, et al Global cerebral hypoperfusion in a patient with transient global amnesia. Journal of computer assisted tomography. 2008;32(3):415–7. Epub 2008/06/04. 10.1097/RCT.0b013e3180de5b9b . 18520548

[34] Ryoo I, Kim JH, Kim S, Choi BS, Jung C, Hwang SI. Lesion detectability on diffusion-weighted imaging in transient global amnesia: the influence of imaging timing and magnetic field strength. Neuroradiology. 2012;54(4):329–34. Epub 2011/05/24. 10.1007/s00234-011-0889-4 . 21603902

[35] Park YH, Jang JW, Yang Y, Kim JE, Kim S. Reflections of two parallel pathways between the hippocampus and neocortex in transient global amnesia: a cross-sectional study using DWI and SPECT. PloS one. 2013;8(7):e67447 Epub 2013/07/19. 10.1371/journal.pone.0067447 ; PubMed Central PMCID: PMCPmc3702497. 23861765 PMC3702497

[36] Chung YA, Jeong J, Yang DW, Kang BJ, Kim SH, Chung SK, et al A Tc-99m SPECT study of regional cerebral blood flow in patients with transient global amnesia. NeuroImage. 2009;47(1):50–5. Epub 2008/12/17. 10.1016/j.neuroimage.2008.11.011 ; PubMed Central PMCID: PMCPmc4292902. 19073268 PMC4292902

[37] Kapucu OL, Nobili F, Varrone A, Booij J, Vander Borght T, Nagren K, et al EANM procedure guideline for brain perfusion SPECT using 99mTc-labelled radiopharmaceuticals, version 2. European journal of nuclear medicine and molecular imaging. 2009;36(12):2093–102. Epub 2009/10/20. 10.1007/s00259-009-1266-y . 19838703

[38] Cohen J. A power primer. Psychological bulletin. 1992;112(1):155–9. Epub 1992/07/01. . 19565683 10.1037//0033-2909.112.1.155

[39] Han K, Chao AC, Chang FC, Chung CP, Hsu HY, Sheng WY, et al Obstruction of Venous Drainage Linked to Transient Global Amnesia. PloS one. 2015;10(7):e0132893 Epub 2015/07/15. 10.1371/journal.pone.0132893 ; PubMed Central PMCID: PMCPmc4501814. 26173146 PMC4501814

[40] Cejas C, Cisneros LF, Lagos R, Zuk C, Ameriso SF. Internal jugular vein valve incompetence is highly prevalent in transient global amnesia. Stroke. 2010;41(1):67–71. Epub 2009/11/21. 10.1161/strokeaha.109.566315 . 19926838

[41] Caplan LR. Transient global amnesia and jugular vein incompetence. Stroke. 2010;41(10):e568; author reply e9. Epub 2010/09/04. 10.1161/strokeaha.110.579789 . 20814012

[42] Modabbernia A, Taslimi S, Ashrafi M, Modabbernia MJ, Hu HH. Internal jugular vein reflux in patients with transient global amnesia: a meta-analysis of case-control studies. Acta neurologica Belgica. 2012;112(3):237–44. Epub 2012/05/04. 10.1007/s13760-012-0072-7 . 22553002

[43] Bartsch T, Dohring J, Reuter S, Finke C, Rohr A, Brauer H, et al Selective neuronal vulnerability of human hippocampal CA1 neurons: lesion evolution, temporal course, and pattern of hippocampal damage in diffusion-weighted MR imaging. Journal of cerebral blood flow and metabolism: official journal of the International Society of Cerebral Blood Flow and Metabolism. 2015;35(11):1836–45. Epub 2015/06/18. 10.1038/jcbfm.2015.137 ; PubMed Central PMCID: PMCPmc4635239. 26082014 PMC4635239

[44] Libby LA, Ekstrom AD, Ragland JD, Ranganath C. Differential connectivity of perirhinal and parahippocampal cortices within human hippocampal subregions revealed by high-resolution functional imaging. The Journal of neuroscience: the official journal of the Society for Neuroscience. 2012;32(19):6550–60. Epub 2012/05/11. 10.1523/jneurosci.3711-11.2012 ; PubMed Central PMCID: PMCPmc3374643. 22573677 PMC3374643

[45] Park YH, Jeong HY, Jang JW, Park SY, Lim JS, Kim JY, et al Disruption of the Posterior Medial Network during the Acute Stage of Transient Global Amnesia: A Preliminary Study. Clinical EEG and neuroscience. 2014 Epub 2014/11/14. 10.1177/1550059414543684 . 25392008

[46] La Joie R, Landeau B, Perrotin A, Bejanin A, Egret S, Pelerin A, et al Intrinsic connectivity identifies the hippocampus as a main crossroad between Alzheimer's and semantic dementia-targeted networks. Neuron. 2014;81(6):1417–28. Epub 2014/03/25. 10.1016/j.neuron.2014.01.026 . 24656258

[47] Ranganath C, Ritchey M. Two cortical systems for memory-guided behaviour. Nature reviews Neuroscience. 2012;13(10):713–26. Epub 2012/09/21. 10.1038/nrn3338 . 22992647

[48] Ritchey M, Libby LA, Ranganath C. Cortico-hippocampal systems involved in memory and cognition: the PMAT framework. Progress in brain research. 2015;219:45–64. Epub 2015/06/15. 10.1016/bs.pbr.2015.04.001 . 26072233

[49] Eichenbaum H, Yonelinas AP, Ranganath C. The medial temporal lobe and recognition memory. Annual review of neuroscience. 2007;30:123–52. Epub 2007/04/10. 10.1146/annurev.neuro.30.051606.094328 ; PubMed Central PMCID: PMCPmc2064941. 17417939 PMC2064941

[50] Spaniol J, Davidson PS, Kim AS, Han H, Moscovitch M, Grady CL. Event-related fMRI studies of episodic encoding and retrieval: meta-analyses using activation likelihood estimation. Neuropsychologia. 2009;47(8):1765–79. 19428409 10.1016/j.neuropsychologia.2009.02.028

[51] Spreng RN, Mar RA, Kim AS. The common neural basis of autobiographical memory, prospection, navigation, theory of mind, and the default mode: a quantitative meta-analysis. Journal of cognitive neuroscience. 2009;21(3):489–510. 10.1162/jocn.2008.21029 18510452

[52] Moon Y, Moon WJ, Han SH. The structural connectivity of the recurrent transient global amnesia. Acta Neurol Scand. 2015 Epub 2015/10/16. 10.1111/ane.12518 . 26467990

[53] Peer M, Nitzan M, Goldberg I, Katz J, Gomori JM, Ben-Hur T, et al Reversible functional connectivity disturbances during transient global amnesia. Annals of neurology. 2014;75(5):634–43. 10.1002/ana.24137 24623317

[54] Kwon Y, Yang Y, Jang JW, Park YH, Kim J, Park SH, et al Left dominance of EEG abnormalities in patients with transient global amnesia. Seizure. 2014;23(10):825–9. Epub 2014/07/20. 10.1016/j.seizure.2014.06.014 . 25037277

[55] Forster A, Al-Zghloul M, Kerl HU, Bohme J, Murle B, Groden C. Value of dynamic susceptibility contrast perfusion MRI in the acute phase of transient global amnesia. PloS one. 2015;10(3):e0122537 Epub 2015/03/25. 10.1371/journal.pone.0122537 ; PubMed Central PMCID: PMCPmc4372367. 25803440 PMC4372367

[56] Forman SD, Cohen JD, Fitzgerald M, Eddy WF, Mintun MA, Noll DC. Improved assessment of significant activation in functional magnetic resonance imaging (fMRI): use of a cluster-size threshold. Magnetic resonance in medicine: official journal of the Society of Magnetic Resonance in Medicine / Society of Magnetic Resonance in Medicine. 1995;33(5):636–47. Epub 1995/05/01. . 7596267 10.1002/mrm.1910330508

[57] Stillhard G, Landis T, Schiess R, Regard M, Sialer G. Bitemporal hypoperfusion in transient global amnesia: 99m-Tc-HM-PAO SPECT and neuropsychological findings during and after an attack. Journal of neurology, neurosurgery, and psychiatry. 1990;53(4):339–42. Epub 1990/04/01. ; PubMed Central PMCID: PMCPmc1014174. 2341849 10.1136/jnnp.53.4.339 PMC1014174

[58] Lin KN, Liu RS, Yeh TP, Wang SJ, Liu HC. Posterior ischemia during an attack of transient global amnesia. Stroke. 1993;24(7):1093–5. Epub 1993/07/01. . 8322387 10.1161/01.str.24.7.1093

[59] Sakashita Y, Kanai M, Sugimoto T, Taki S, Takamori M. Changes in cerebral blood flow and vasoreactivity in response to acetazolamide in patients with transient global amnesia. Journal of neurology, neurosurgery, and psychiatry. 1997;63(5):605–10. Epub 1998/01/04. ; PubMed Central PMCID: PMCPmc2169813. 9408101 10.1136/jnnp.63.5.605 PMC2169813

[60] Takeuchi R, Yonekura Y, Matsuda H, Nishimura Y, Tanaka H, Ohta H, et al Resting and acetazolamide-challenged technetium-99m-ECD SPECT in transient global amnesia. Journal of nuclear medicine: official publication, Society of Nuclear Medicine. 1998;39(8):1360–2. Epub 1998/08/26. . 9708507

[61] Nardone R, Buffone EC, Matullo MF, Tezzon F. Motor cortex excitability in transient global amnesia. Journal of neurology. 2004;251(1):42–6. Epub 2004/03/05. 10.1007/s00415-004-0270-1 . 14999488

